# Effect of Brivaracetam on Efficacy and Tolerability in Patients With Brain Tumor-Related Epilepsy: A Retrospective Multicenter Study

**DOI:** 10.3389/fneur.2020.00813

**Published:** 2020-08-19

**Authors:** Marta Maschio, Andrea Maialetti, Cristina Mocellini, Elisabetta Domina, Giada Pauletto, Cinzia Costa, Addolorata Mascia, Michele Romoli, Diana Giannarelli

**Affiliations:** ^1^Center for Tumor-Related Epilepsy, UOSD Neurology, Regina Elena National Cancer Institute IRCCS IFO, Rome, Italy; ^2^UO Neurology ASO S. Croce e Carle, Cuneo, Italy; ^3^U.C. Neurology, Ospedale Maggiore di Lodi ASST, Lodi, Italy; ^4^Neurology Unit, Azienda Sanitaria Universitaria, ASUFC, Udine, Italy; ^5^Clinic of Neurology, Ospedale SM Misericordia, Università degli Studi di Perugia, Perugia, Italy; ^6^Center for Epilepsy Surgery, IRCCS Neuromed, Pozzilli, Italy; ^7^Biostatistic Unit, Regina Elena National Cancer Institute IRCCS IFO, Rome, Italy

**Keywords:** brain tumor-related epilepsy, antiseizure medication, glioma, responder rate, brivaracetam, adverse events

## Abstract

**Background:** Epilepsy is a common symptom of brain tumors and is often pharmacoresistent. Among new antiseizure medications (ASMs) Brivaracetam (BRV) has been approved as adjunctive treatment for focal seizures and it was tested in non-oncological patient populations. This is the first study that retrospectively explored efficacy and tolerability of BRV as add-on therapy in brain tumor-related epilepsy (BTRE) patients.

**Materials and Methods:** We reviewed the medical records of 33 BTRE patients from six Italian epilepsy centers; charts included tumor history, diagnosis of BTRE, BRV added as first or second add-on for uncontrolled seizures and/or adverse events (AEs) of the previous ASMs, at least 1-month follow-up, seizure frequency, and AEs assessment.

**Results:** Thirty-three patients (19 males, mean age: 57.6 years; 14 females, mean age: 42.4 years): 11 low grade gliomas, five high grade gliomas, six meningiomas, 10 glioblastomas, one primary cerebral lymphoma. Fourteen patients had focal aware seizures, nine focal unaware, seven focal to bilateral tonic-clonic seizures, three patients presented more than one seizure type: focal unaware with focal to bilateral tonic clonic seizures (two patients) and focal aware and unaware seizures (one patient). Mean seizure frequency in the month preceding BRV introduction: 7.0; at last follow-up: 2.0 (*p* = 0.001). Seven patients (21.2%) reported AEs (anxiety, agitation, fatigue, vertigo) and three of them (9.0%) required drug withdrawal due to psychiatric adverse events (PAEs). Three other patients withdrew BRV: one for scarce compliance (3.0%), two for uncontrolled seizures (6.0%).

**Conclusion:** Our results showed that BRV could be a new therapeutic option effective in reducing seizures in BTRE patients, taking into account the incidence of PAEs in this particular population. Future and larger prospective studies are needed.

## Introduction

Epilepsy is one of the most common symptoms of brain tumors and it is often pharmacoresistent (1).

The selection of the appropriate antiseizure medication (ASM) in brain tumor related epilepsy patients (BTRE) should be driven by multiple factors, which include not only efficacy in the specific type of seizure to be treated but also tolerability and drug-interaction potential (2).

Among new ASMs, brivaracetam (BRV) is a 2-pyrrolidinone derivative that has been approved as adjunctive therapy and monotherapy for focal (partial-onset) seizures in patients with epilepsy in United States and as adjunctive therapy for focal seizures in patients with epilepsy in the European Union (3).

BRV is an analog of Levetiracetam (LEV) and also selectively binds a novel brain specific binding site synaptic vesicles protein 2A (SV_2A_). In addition, BVR inhibits voltage dependent Na^+^ currents and reverses the inhibitory effect of negative allosteric modulators of aminobutyric acid (GABA) and glycine induced current (4, 5).

BRV presents a favorable pharmacokinetic profile, linear and predictable, with low intersubjective variability and almost 100% bioavailability (6). BRV is extensively metabolized through several metabolic pathways and is fully excreted by urine (only 8–11% remains unchanged). The efficacy of BRV as add-on therapy in non-oncological patients with uncontrolled focal seizures has been assessed in six randomized placebo-controlled trials (7–12). The most common adverse events (AEs) reported in literature include: fatigue, dizziness, somnolence, which apparently disappear during treatment (8); moreover a low incidence of neurobehavioral and cognitive AEs is reported (8, 13).

To date, there are no data on efficacy and tolerability of BRV in BTRE patients. However, the choice of antiseizure treatment in this patient population must also take into account the potential interactions between ASMs and chemotherapy and/or support therapies (i.e., corticosteroids) which can induce AEs that may be more frequent than in non-oncological epileptic population (14, 15). Moreover, in this patient population, AEs can affect patient's quality of life more than seizure frequency (16).

Finally, our previous study showed that BRV *in vitro* exerts a dose-dependent cytotoxic effect on various glioma cell lines, and this effect was concomitant with the modulation of a number of micro RNA (miRNAs) (17), which has been identified in previous studies as predictive marker of seizure occurrence (18) and tumor progression (19).

The aim of this study is to retrospectively evaluate efficacy and tolerability of BRV as add-on therapy, in BTRE patients treated for at least 1 month in six different Italian Epilepsy Centers.

## Materials and Methods

### Concept Design

Retrospective study (RS 1332/20, 24/04/2020) on medical charts from six Italian Epilepsy Centers of BTRE patients treated for at least 1 month with brivaracetam as add-on in adherence to current clinical practice (GU 31.03.2008, Determinazione AIFA 20.03.2008). Each center was required to send anonymized data regarding BTRE patients seen from September 2018 to February 2019 and followed for at least 1 month. The caring physician had to record and date the patients' clinical record of all actions taken during follow-up (with particular reference to changes in ASM therapy for ineffectiveness and/or adverse effects and concurrent therapies).

The participating centers adhered to the standard follow-up of BTRE patients, and ASM treatment was chosen based on the guidelines of the International League Against Epilepsy (ILAE) (20). All data were collected and merged through an anonymous Excel file developed and agreed upon by the participating centers. Control of quality and completeness of collected data were performed before the statistical analyses. Centers were requested to answer specific queries in the event that further clarification was necessary and, to reduce selection bias, all patients present in the centers' archives were screened and all consecutive patients fulfilling the selection criteria were enrolled.

Medical charts had to include the following information: diagnosis of primary brain tumor according to World Health Organization (WHO) (21); type of surgery: biopsy or surgical resection (partial/total); presence of chemotherapy (CT), radiotherapy (RT) and/or corticosteroids before or during the follow-up period: yes or no; diagnosis of structural epilepsy and seizure classification (focal: aware/unaware) according to new ILAE classification (20); type and dosage of ASMs; BRV added as first or second add-on for uncontrolled seizures and/or for adverse effects of previous ASMs; number of seizures in the month preceding BRV introduction (one or more than one seizure per month) and during follow-up; AEs occurred during BRV therapy collected by patients' spontaneous report (22); date of last follow-up.

An “adverse event” (AE) was defined as any unfavorable and unintended sign, symptom, or disease temporally associated with the medical treatment. Symptoms related to tumor progression were not considered to be AEs. All AEs were recorded in our database, and an AE was attributed to a specific ASM if the attending physician had already written in the medical chart that it had to be attributed directly to the drug or if the AE only occurred or aggravated after starting or increasing the dose of ASM. We defined intolerable AE an AE that led to a decrease in dose or cessation of an ASM. We categorized AEs according NCI-CTCAE as: psychiatric (PAEs) (sedation, agitation, anxiety, irritability), central nervous system (CNSAEs) (vertigo, fatigue), and defined their severity according to NCI-CTCAE classification as grade 1–5 (mild, moderate, severe, life threatening consequences, death) (23).

#### Primary Aim

To retrospectively evaluate efficacy on seizure control and tolerability of BRV as add-on therapy in BTRE patients.

#### Secondary Aim

To retrospectively detect the incidence of BRV-related side effects during follow-up period compared with baseline.

#### Primary Efficacy Variable

Efficacy of BRV was assessed comparing mean seizure frequency at basal visit and at last follow-up available, after each patient reached minimal effective dose of 50 mg/die.

#### Secondary Efficacy Variables

BRV related side effects at last follow-up available compared to baseline.

### Statistical Methods

We computed descriptive statistics for all variables of interest. Continuous data were reported as the mean and standard deviation and we represented categorical data with frequencies and percentage values. In order to investigate the relationships between categorical variables, the Pearson's Chi-squared test and the Fisher Exact test were employed as appropriate. For continuous variables Student's *t*-test or Mann-Whitney test were used. All statistical analyses were performed with SPSS statistical software version 20 (SPSS Inc., Chicago IL, USA). It was calculated that 24 patients allowed us to evaluate a mean reduction in monthly seizure frequency at about 60% of the standard deviation, assuming a level of significance of 5% and a power of 80%. For each patient we extrapolated the number of seizure in the months immediately before the introduction of brivaracetam from the medical chart, and we compared it with the monthly seizure frequency until the last observation.

## Results

We reviewed medical charts of 33 BTRE patients (19 males, mean age: 57.6 years; 14 females, mean age: 42.4 years) from six Italian Epilepsy Centers, followed from 2 to 48 months (mean follow-up duration 10 months), between September 2018 and February 2019. Two patients of our sample had a shorter follow-up, for occurrence of PAEs that led to drug's withdrawal (2 months) and for disease progression (3 months).

Eleven patients (33.3%) had low grade glioma (LGG), 5 (15.2%) high grade glioma (HGG), 6 (18.2%) meningiomas (MEN) (including 1 anaplastic meningioma), 10 (30.3%) glioblastoma (GBM), and one (3.0%) primary cerebral lymphoma (LYM). Tumor site was frontal lobe in 13 patients, temporal lobe in 12, parietal lobe in five, and multilobular in three. Six patients (18.2%) were IDH1 mutated; 11 (33.3%) were negative; 16 (48.5%) were unknown. Twelve patients (36.4%) were MGMT metilated; 1 (3.0%) was unmetilated; in 20 patients (60.6%) the methylation status was not known. Twenty patients underwent gross total resection, 10 partial resections, two biopsies, and one did not undergo surgery; during follow-up nine, patients underwent chemotherapy and one patient underwent radiotherapy (see Table 1). Disease progression during BRV treatment indicated by available neuroradiological data was observed in 11 patients (33.3%).

**Table 1:** Patients' clinical and vital data.

**Pat**	**Age (range)**	**Sex**	**Histology**	**Site of tumor**	**Surgery**	**Chemotherapy**	**RT**	**Seizure type**	**No. of seizures in the month before entering the study**	**Months of follow-up available**	**ASMs before BRV**	**ASMs during BRV**	**BRV dose assigned (mg/day)**	**No. of seizures/month at last follow-up available**	**Adverse events during BRV therapy**	**Disease progression during BRV follow-up**	**Drop out**
1	45-49	F	MEN	Parietal	GTR	No	Yes°	F-B T-C	8.0	2.0	LCM 400+VPA 800+PB 150	LCM 300+VPA 800+PB 100	100	5,0	Agitation	No	Yes
2	40-44	F	GBM	Temporal	GTR	BEV+TMZ+ OTHER*	Yes°	FA	2.0	12.0	CBZ 400+LTG 400+CLZ 14	LTG 400+CLZ 14	100	2,0	No	Yes	No
3	50-54	M	LGG	Multilobular	PR	TMZ°	Yes°	F-U+F-B T-C	10.0	48.0	LEV 3000+LCM 400	LCM 400	200	3,0	No	No	No
4	50-54	F	GBM	Multilobular	PR	TMZ§	Yes§	FA+FU	30.0	6.0	LEV 1500+LCM 100	LCM 100	100	0,0	No	No	No
5	75-79	F	GBM	Frontal	PR	TMZ°	Yes°	F-U+F-B T-C	4.0	8.0	LEV 2000	CLZ 20	150	4,0	Fatigue	Yes	No
6	45-49	F	LGG	Temporal	GTR	No	No	FA	3.0	6.0	ZNS 125	ZNS 125	100	0,0	No	No	No
7	30-34	M	LGG	Temporal	PR	No	Yes°	F-B T-C	28.0	11.0	LCM 200+LEV 3000	LCM 200	200	4,0	Fatigue	No	No
8	35-39	F	HGG	Temporal	GTR	TMZ*	Yes°	FA	4.0	10.0	LCM 300	LCM 200	100	0,0	Vertigo	No	No
9	60-64	F	GBM	Temporal	GTR	FTM+TMZ°	Yes°	F-B T-C	8.0	10.0	LCM 200+PRP 8+LEV 1500	LCM 200+PRP 8	200	8,0	Anxiety	No	Yes
10	35-39	F	LGG	Multilobular	PR	No	Yes°	F-B T-C	3.0	8.0	LCM 200	LCM 200	100	0	No	No	No
11	45-49	M	HGG	Parietal	GTR	TMZ°	Yes°	FA	15.0	6.0	PRP 6+LEV 2000	LCM 100+ PRP 2	150	0	Anxiety+Agitation	Yes	No
12	55-59	M	MEN	Temporal	GTR	No	No	F-B T-C	10.0	6.0	ZNS 300+LEV3000+LCM 400+PRP 8	ZNS 300+LCM 400+PRP 8	100	15.0	Uncontrolled seizures	No	Yes
13	35-39	M	GBM	Frontal	PR	TMZ°	Yes°	F-B T-C	2.0	12.0	LEV 2000+LCM 100	LCM 200	200	1,0	No	Yes	No
14	60-64	M	A-MEN	Frontal	GTR	No	Yes°	FA	10.0	12.0	CBZ 600+LEV 1500	CBZ 200	200	0,0	No	No	No
15	35-39	M	LGG	Temporal	PR	No	No	FU	10.0	24.0	CBZ1200+ZNS 250	CBZ 1200	200	3,0	No	No	No
16	40-44	F	LGG	Parietal	GTR	No	No	FU	10.0	30.0	LEV 2500+ZNS 200	ZNS 200	200	0	No	No	No
17	50-54	M	HGG	Frontal	PR	TMZ°	Yes°	FA	0	18.0	LEV 3000+LCM 200	LCM 200	200	0	No	No	No
18	70-74	M	LGG	Temporal	BIO	No	Yes°	FU	30.0	12.0	VPA 1000	VPA 500	200	3	No	No	No
19	20-24	M	GBM	Frontal	GTR	TMZ+FTM*	Yes°	FA	2.0	18.0	LEV 1500	CLZ 10	200	0	No	No	No
20	50-54	F	GBM	Parietal	PR	TMZ°	Yes°	FA	2.0	14.0	LEV 2500+LCM 200	LCM 200	200	0	No	No	No
21	40-44	F	LGG	Frontal	GTR	TMZ+OTHER*	Yes°	FA	2.0	2.0	LEV 1500+ LCM 150	LCM 150	200	8	Uncontrolled seizures	Yes	Yes
22	60-64	M	LGG	Temporal	No	No	No	FU	1.0	36.0	CBZ 600	CBZ 200	200	0	No	No	No
23	20-24	M	LGG	Temporal	PR	No	No	FU	4.0	10,0	CBZ 800	CBZ 800	200	5,0	No	No	Yes
24	45-49	M	GBM	Parietal	GTR	TMZ+FTM*	Yes°	FU	6.0	5.0	LCM 400	LCM 400	150	0,0	No	Yes	No
25	40-44	F	MEN	Frontal	GTR	No	No	FU	3.0	6.0	OXC 600	OXC 600	200	0,0	Anxiety	No	Yes
26	70-74	M	LYM	Frontal	GTR	OTHER°	No	FA	3.0	6.0	LEV 2000+ CLZ 20	CLZ 20	200	0,0	No	No	No
27	45-49	M	HGG	Frontal	GTR	TMZ+FTM*	Yes°	FA	6.0	11.0	OXC 1200+CLZ 10	OXC 1200+CLZ 10	200	1	No	Yes	No
28	75-79	F	MEN	Frontal	GTR	No	No	F-B T-C	2.0	12.0	LEV 2000+ CLZ 10	CLZ 10	200	0	No	No	No
20	70-74	M	LGG	Temporal	GTR	TMZ°	No	FA	3.0	8.0	LEV 2000+ CLZ 10	CLZ 10	200	0	No	No	No
30	65-69	M	MEN	Frontal	GTR	No	No	FU	3.0	5.0	LEV 2000+ CLZ 20	CLZ 20	200	0	No	Yes	No
31	60-64	M	HGG	Temporal	BIO	TMZ°	No	FA	3.0	6.0	VPA 1500	VPA 1500	200	0	No	Yes	No
32	65-69	M	GBM	Frontal	GTR	TMZ+FTM*	Yes°	FU	4.0	3.0	LEV 2000+ CLZ 20	CLZ 20	200	0	No	Yes	No
33	75-79	F	GBM	Frontal	GTR	TMZ+FTM*	Yes°	FA	2.0	6.0	LEV 2000+ CLZ 10	CLZ 10	200	0	No	Yes	No

*Histology: LGG, low grade glioma; HGG, high grade glioma; MEN, meningioma; A-MEN, anaplastic meningioma; GBM, glioblastoma; LYM, primary cerebral lymphoma*.

*-Surgery: PR, partial resection; GTR, gross total resection*.

-Chemotherapy: °before; *before and during follow-up; ^§^during follow-up.

*-Type of chemotherapy: TMZ, temozolomide; FTM, fotemustine; BEV, bevacizumab*.

-RT, Radiotherapy: °before; *before and during follow-up; ^§^during follow-up.

*-Seizure type: FA, focal aware seizures; FU, focal unaware seizures; F-B T-C, focal to bilateral tonic-clonic*.

*-ASMs (antiseizure medications): LEV, levetiracetam; VPA, valproic acid; CBZ, carbamazepine; OXC, oxcarbazepine; LTG, lamotrigine; TPM, topiramate; ZNS, zonisamide; LCM, lacosamide; PRP, perampanel; CLZ, clobazam*.

Fourteen patients (42.4%) had focal aware seizures, 9 (27.3%) focal unaware, 7 (21.2%) focal to bilateral tonic-clonic seizures, three patients presented more than one seizure type: focal unaware with focal to bilateral tonic clonic seizures (two patients; 6.06%) and focal aware and unaware seizures (one patient; 3.03%). Before starting BRV treatment, 11 patients (33.3%) were on ASM monotherapy (valproic acid-VPA: two patients; carbamazepine-CBZ: two patients; oxcarbazepine-OXC: one patient; zonisamide- ZNS: one patient; levetiracetam-LEV: two patients; lacosamide-LCM: three patients); and 22 (66.6%) on polytherapy (see Table 1).

BRV was introduced for AEs of previous ASMs in five patients (15.2%), for uncontrolled seizure in 19 (57.6%), and for both reasons in 9 (27.3%). BRV starting dosage was 25 mg/die; mean dosage at final follow-up was 175 mg/die.

All patients (*n* = 20) who assumed LEV in mono or polytherapy were switched to BRV.

During BRV therapy 27 patients (81.8%) assumed one ASM in addiction to BRV (valproic acid-VPA: two patients; carbamazepine-CBZ: four patients; oxcarbazepine-OXC: one patient; zonisamide- ZNS: two patients; clobazam-CLZ: eight patients; lacosamide-LCM: 10 patients) and six patients (18.1%) assumed more than one ASM in addition to BRV (see Table 1).

**Figure 1:**
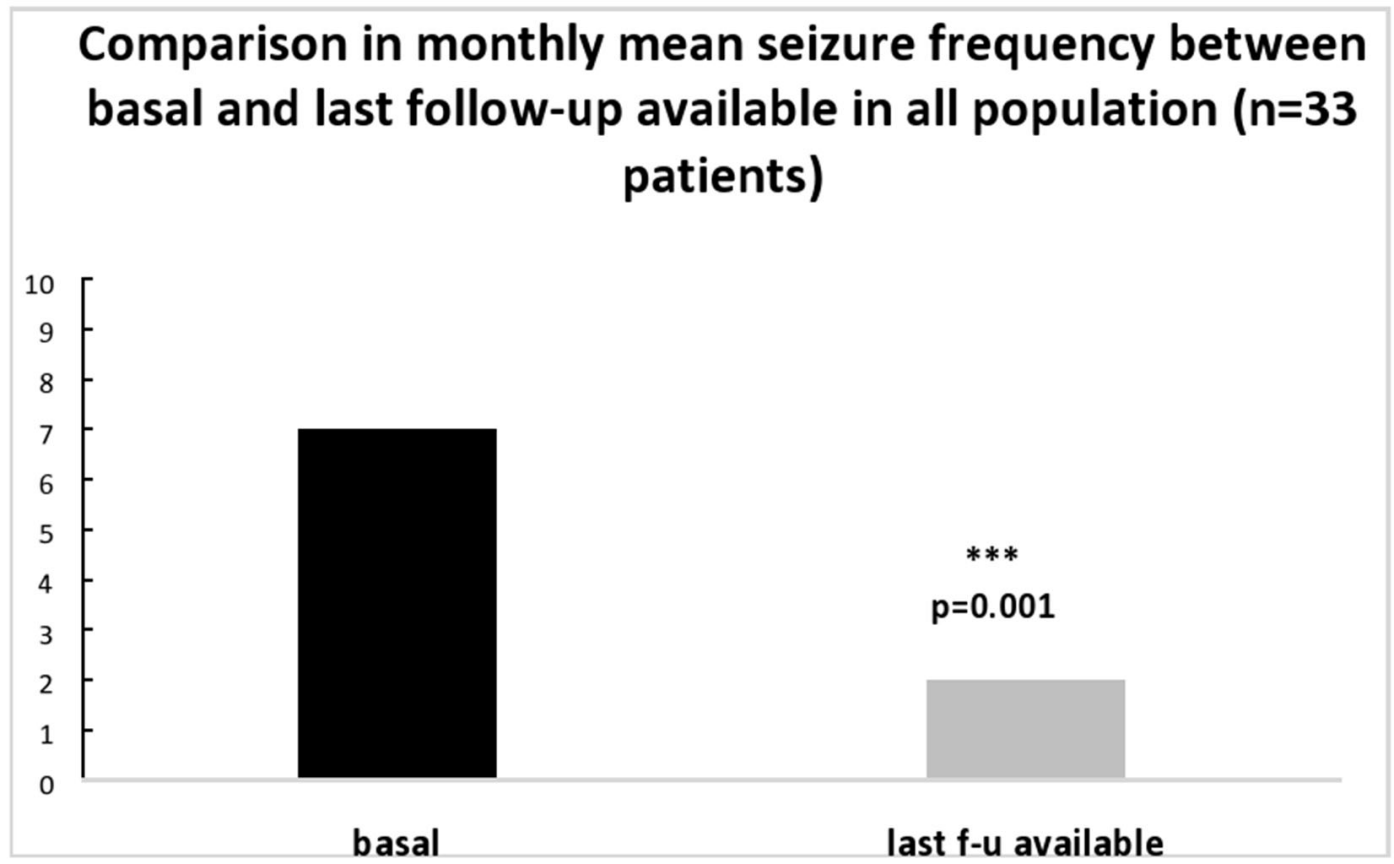
Comparison in mean seizure number/month between basal and at last follow-up available in all population (*n* = 33 patients).

After a mean follow-up of 10 months (duration between 2 and 48 months), monthly mean (±SD) seizure frequency significantly decreased from 7.0 ± 7.9 at basal to 2.0 ± 3.6 at last follow-up available (*p* = 0.001) (see Figure 1). Twenty patients were seizure free (60.6%), 6 had a reduction ≥50%, one had a reduction ≤50%, 3 were unchanged, 2 patients had an increase in monthly seizure frequency and returned to previous ASM, and one patient shifted to previous ASM due to scarce compliance. The responder rate was 78.7%.

We further analyzed differences in BRV efficacy in patients who were switched from LEV to BRV (LEV group; *n* = 20) vs. patients who did not assume LEV as previous therapy (non-LEV group, *n* = 13).

We observe similar response at BRV: mean monthly seizure frequency significantly decreased at last follow-up available compared to basal evaluation in both groups (LEV group from 7.5 ± 8.4 to 2.3 ± 4.5; *p* = 0.01; non-LEV group from 6.4 ± 7.5 to 1.5 ± 1.9; *p* = 0.03).

Regarding the evaluation of AEs, seven patients (21.2%) out of 33 reported AEs during BRV treatment such as: anxiety (two patients), agitation (one patient), anxiety and agitation (one patient), fatigue (two patients), vertigo (one patient). Among these, patients who experienced PAEs: anxiety (2) and agitation (1) required drug discontinuation (9.0%); one patient (3.0%) with anxiety and agitation had dose reduction with a gradual return to pre-drug conditions; the remaining 3 patients (9.0%) who experienced CNSAEs, such as vertigo or fatigue, ameliorated spontaneously (see Table 2).

**Table 2:** Adverse events (AEs) reported in medical charts during BRV treatment.

	**Number of patients**	**%**	**Action taken**
Anxiety	2	6.0	Drug's discontinuation
Agitation	1	3.0	Drug's discontinuation
Anxiety+Agitation	1	3.0	Dose reduction
Vertigo	1	3.0	None
Fatigue	2	6.0	None

We also analyzed whether the incidence of AEs was different in patients who assumed LEV in mono or polytherapy as previous treatment compared to those who did not assumed LEV. We did not observe any significant difference regarding the appearance of BRV related AEs between 20 patients who switched from LEV to BRV (four patients experienced AEs; 20%) and 13 patients without LEV as previous therapy (three patients experienced AEs; 23.0%) (*p* = 0.78).

In order to assess if BRV efficacy could be influenced by factors related to tumor disease, we compared the number of patients seizure-free vs. patients non seizure-free, in different oncological situations such as: different histology (LGG/HGG; *p* = 0.28), tumor localization (frontal/temporal/parietal/multilobular; *p* = 0.16), type of surgery (gross total resection/partial resection; *p* = 0.11), chemotherapy (yes/no; *p* = 0.28), radiotherapy (yes/no; *p* = 0.20), stage of disease (disease progression/stable disease; *p* = 0.61), IDH1 mutated and non-mutated (*p* = 0.90); all correlations were not significant. Comparison between patients MGMT-metilated and non-metilated was not evaluable due to the small sample size (12 patients vs. 1 patient).

## Discussion

Among the new generation ASMs, BRV is a new therapeutic option in the treatment of drug-resistant epilepsy in adult patients. The drug was tested as adjunctive therapy in different trials only in non-oncological patient populations, from which emerged a good efficacy with a favorable safety profile (7–12, 24–27). This is the first study that explored the efficacy and tolerability of BRV in brain tumor-related epilepsy patients.

We reported the results of retrospective analysis on medical charts of 33 BTRE patients treated with BRV in add-on and followed for a mean of 10 months, between September 2018 and February 2019. BRV treatment was associated with a significant reduction in mean monthly seizure frequency, which decreased from 7.0 ± 7.9 at baseline to 2.0 ± 3.6 at last follow-up available. Responder rate was 78.7% with 20 patients (60.6%) seizure free and 6 patients (18.1%) with a seizure reduction ≥50%. Monthly seizure frequency remained stable in 3 patients and worsened in 2. Literature data from real-life experience studies in non-oncological patients populations with refractory partial epilepsy reported a good efficacy of BRV (25, 26). Steinhoff et al., in a retrospective study on 101 patients treated with BRV in add-on, observed after 6 months of treatment a responder rate of 27.8% with 7% of patients seizure free. Villanueva and colleagues, in a multicenter retrospective analysis on 575 patients, reported a mean reduction in seizure frequency of 36% at 12 months follow-up, with 39.7% of responders and 17.5% of seizure free patients. Even a systematic review and meta-analysis (28) highlighted the effectiveness of BRV as add-on in reducing seizure frequency in adults with drug-refractory non-oncological focal epilepsy. Our results confirm for the first time a good efficacy of BRV in add-on also in BTRE patients, with a high percentage of responders.

Moreover, literature data on adults with drug-refractory non-oncological focal epilepsy (28) reported a greater BRV related-treatment effect in LEV-naïve patients, rather than in patients who previously assumed LEV, in which BRV showed lower efficacy. In our population with BTRE, we observed similar response to BRV both in the sub-group of patients switched from LEV and in the sub-group of patients who did not assume LEV as previous therapy. Regarding BRV tolerability, our results showed the incidence of AEs in 7 patients (21.2%), which consisted of: agitation, anxiety, fatigue, and vertigo. PAEs observed in 4 patients (57.1%) were the main reason for drug discontinuation (2 patients for anxiety, 1 patient for agitation) or reduction (1 patient for anxiety and agitation), while CNSAEs such as vertigo or fatigue (observed in 3 patients) ameliorated spontaneously during treatment. Previous studies (25, 26) on tolerability of BRV in non-oncological patient population reported the presence of AEs such as: dizziness, somnolence, irritability, anxiety, aggression, and depression. Villanueva and colleagues observed an incidence of physical and psychiatric AEs, respectively in 39.8 and 14.3% of cases; the highest percentage of drug discontinuation was due to physical side effects (8.9%). In a retrospective study by Steinhoff et al., incidence of AEs was about 37%, most of which were dizziness and somnolence, with a lower rate of psychiatric ones; the main reason for drug discontinuation was lack of efficacy.

Our results showed in our patients with BTRE a total number of AEs lower than in non-oncological patients. Nevertheless, the incidence of PAEs was higher while the incidence of CNSAEs was not. For this reason, we recommend clinicians to inform the caregivers and patients of possible AEs upon initiating ASMs for BTRE, carefully monitoring their incidence and considering change of therapy if AEs reduce patients' quality of life. Furthermore, in our sample, the appearance of BRV related AEs was not affected by assuming LEV as previous therapy. We did not observe significant differences in AEs occurrence between patients who were switched from LEV to BRV and patients in whom BRV was added, as previously reported by Steinhoff et al. (25), in non-oncological patients population.

Finally, literature data on BTRE patients (29) indicated that a number of factors related to oncological disease (i.e., histology, tumor location, type of surgery, molecular markers) have been associated with a higher seizure risk, but, in our patient, BRV as add-on therapy maintained a good efficacy over time independently by these risk factors.

This study has several limitations. First, this is a retrospective study. Data have been obtained from medical records where, in the absence of standardized and systematic collection of the required information, variables were available in non-standardized format and occasional variables, not directly correlated with the aim of the study, were excluded.

Second, treatment retention was assessed in an observational context. Physicians' and patients' judgment might have had strong influence on the decision to start/stop the assigned treatment. Third, this is a multicenter study. Management of the disease varies across centers and this may have a strong impact on study results. Finally, the small sample size and the relatively short follow-up could have influenced our results. Regarding to this aspect, it has to be considered that patients with epilepsy and brain tumors represent a very fragile population, with a complex clinical profile, poor life expectancy, and a rare disease. For this reason, it was difficult to balance the need to have a sufficiently large sample of patients, with the need to have a statistically homogenous sample size with long follow-up.

All these limitations imply a cautious interpretation of our findings and should encourage future multicenter studies with randomized trial and longer follow-up aimed to further evaluate the role of BRV as add-on therapy in BTRE patient populations.

## Conclusion

Despite the limitations of our retrospective study, our results showed that BRV could be a new therapeutic option effective in reducing epileptic seizures in BTRE patients, taking into account the incidence of psychiatric adverse events in this particular patient population.

## Data Availability Statement

The datasets presented in this study can be found in online repositories. The names of the repository/repositories and accession number(s) can be found in the article/supplementary material.

## Ethics Statement

The studies involving human participants were reviewed and approved by Ethical Committee RS 1332/20, 24/04/2020. Written informed consent for participation was not required for this study in accordance with the national legislation and the institutional requirements.

## Author Contributions

MM: conceptualization and methodology, project administration and supervision, data collection, and writing-review and editing. AMai: data collection and writing-original draft. CM, ED, GP, CC, AMas, and MR: data collection. DG: statistical analysis. All authors contributed to the article and approved the submitted version.

## Conflict of Interest

MM has received support for travel to congresses from EISAI srl; has participated in scientific advisory boards for EISAI; has participated in pharmaceutical industry-sponsored symposia for UCB Pharma; has received research grants from UCB Pharma. GP has participated in advisory boards and industry-sponsored symposia for Eisai, UCB, LivaNova. She received speakers' fees from Eisai, LivaNova and UCB in the past 3 years. CC has received research grants from UCB and EISAI; has participated in scientific advisory boards for EISAI; has participated in pharmaceutical industry-sponsored symposia for EISAI. The remaining authors declare that the research was conducted in the absence of any commercial or financial relationships that could be construed as a potential conflict of interest.
